# An unexpected case of an adnexal hydatid cyst in a pregnant woman: a case report

**DOI:** 10.1186/s13256-024-04640-x

**Published:** 2024-07-07

**Authors:** Ana-Maria Brezeanu, Dragoș Brezeanu, Vlad-Iustin Tica

**Affiliations:** 1 Ovidius University of Constanta, Constanta, Romania; 2 County Clinical Emergency Hospital “Sf. Ap. Andrei”, Constanta, Romania

**Keywords:** Echinococcosis, Zoonosis, Pregnancy, Adnexal tumor, Case report

## Abstract

**Background:**

Cystic echinococcosis, also known as hydatid disease, is a chronic and endemic illness caused by infection with a parasite called *Echinococcus granulosus*. In Romania, this disease has an incidence rate of 5.6 per 100,000 individuals, which is the highest in the Dobrogea region. The liver is the most affected site, accounting for 68.8% of cases, followed by the lungs at 17.2%. While cases of hydatid disease in the genital organs are rare, occurring at an incidence rate of only 0.5%, it is worth noting that cases of this disease in pregnancy worldwide are also rare, occurring at an incidence rate of 1 in 20,000 to 1 in 30,000 pregnancies.

**Case report:**

A 15-year-old Eastern-European woman who was 12 weeks pregnant presented to the emergency room with acute pelvic pain, dysuria, and frequent urination. Her laboratory tests showed that she had a urinary tract infection, and pelvic ultrasound revealed that she had a mass on her right adnexa. Despite receiving treatment, her symptoms did not improve, and she had to undergo surgery to remove the mass, which turned out to be a hydatic cyst. She also had to undergo a cesarean section to deliver her baby owing to fetal distress during labor.

**Conclusions:**

This medical case report provides a detailed description of a pelvic hydatid cyst that was discovered during pregnancy. What makes this case particularly noteworthy is the cyst’s unusual location—it was found at the level of the right broad ligament of the uterus. Despite the complexity of the situation, the patient was able to receive effective treatment and the cyst was successfully managed with great outcomes for both the patient and the newborn. We hope that this report serves as a valuable example of how medical professionals can navigate challenging cases and provide optimal care for their patients.

## Background

Cystic echinococcosis is a chronic endemic disease caused by infection with a parasite called *Echinococcus granulosus*, a cestode whose life cycle involves dogs as definitive hosts [1]. Adult worms usually live in the intestines of dogs but can also be found in goats, sheep, and pigs as intermediate hosts. Four species of *Echinococcus* raise public health concerns, although six taeniid cestodes from this genus are known [2]. Multiple genotypes of *Echinococcus granulosus* were identified, and they differ in their distribution, host range, and morphological features [3].

According to the World Health Organization, the incidence is about 50 per 100,000 persons with a prevalence rate of 5–10%, especially in endemic countries. On the other hand, the prevalence of the disease in animals in hyperendemic areas is much higher; for example, in South America, it varies from 20% to 95% [4]. Nonetheless, the incidence of cystic echinococcosis in Romania is around 5.6 per 100,000 persons, with the highest incidence in the Dobrogea region [5].

Humans are the intermediate hosts and will acquire the infection by ingesting proglottids or eggs passed from dog feces that contaminate the food or water that people ingest [6]. The exact time for a cyst to develop is about 10 months. Studies showed a wide variation, with up to 40–80% of patients with primary cystic echinococcosis having single-organ involvement and harboring a solitary cyst [7]. The most common site of implantation is the liver (68.8%), followed by lung (17.2%), kidney (3.7%), and spleen (3.3%) [7]. In symptomatic cases, the liver/lung ratio (the most common two implantation sites) is only 2.5:1, based on hospital records, or 4.1:1, based on autopsy records [8]. The occurrence of hydatid disease in the reproductive system is shallow, with an incidence of only 0.5% [9]. Worldwide, the incidence of hydatid disease in pregnancy ranges from 1 in 20,000 to 1 in 30,000 pregnancies.

The management of cystic echinococcosis during pregnancy is challenging because there is no consensus, and each case must be treated individually [10]. During pregnancy, hydatid cysts may enlarge because of the decreased cell-mediated immunity and the humoral effects of placental steroids. There is also an increased risk of cyst rupture or ovarian torsion with consecutive anaphylaxis [11]. A quick and early diagnosis in pregnancy is essential because the cyst can increase rapidly. Symptoms are often absent; imaging studies detect many cysts incidentally [12]. Drugs, such as albendazole and mebendazole, can be used in the second and third trimesters, but surgical therapy remains the primary treatment, although, during pregnancy, it may be difficult [9]. It is important to bear in mind that the existence of an infection and chronic medical issues can have a significant detrimental effect on the prognosis of this intricate problem [13, 14]. 

This case report described a pelvic hydatic cyst during pregnancy, with an unusual localization at the level of the right broad ligament of the uterus.

## Case report

A 15-year-old Eastern-European primigravida presented to the emergency department at 12 weeks gestation with complaints of right flank and lower back pain, pollakiuria, and dysuria. Her past medical history was significant for disseminated hydatid disease, for which she underwent splenectomy, hepatic segmentectomy, and pulmonary lobectomy at the age of 6 years. However, she failed to comply with the prescribed medication and did not seek further follow-up care.

Her physical examination revealed a blood pressure of 117/63 mmHg, a heart rate of 77 beats/minute, and a body mass index of 19.7 kg/m^2^. The temperature was 37.5 ℃. The obstetrical examination revealed a soft abdomen, mild tenderness in the umbilical and suprapubic region, and a mass corresponding to 12–13 weeks of gestation, arising from the pelvis. Giordano’s maneuver was positive on the right lumbar side. There was no sign of abnormal vaginal discharge or vaginal bleeding during vaginal examination.

An abdominal/pelvic ultrasound identified a single live fetus with a biparietal diameter of 26 mm, corresponding to 14 weeks and 4 days. Cardio-fetal beats were 138 beats/minute, the amniotic fluid index was within normal range, and the placenta was located posteriorly. On the right side of the uterus, an adnexal cyst of 12 cm was found. The laboratory workup revealed leukocytosis with 21,690 leucocytes/l, no anemia, and 679,000 thrombocytes/l. The urinalysis revealed that leukocytosis and the urine culture were positive for *Escherichia coli* with sensitivity to cefuroxime. The other infectious analysis, such as human immunodeficiency virus (HIV), syphilis, hepatitis, endocervical culture, and vaginal culture, was negative. The patient was diagnosed with a urinary tract infection and treated with antibiotics with cefuroxime 1 g/12 hours for 7 days.

The workup for the right adnexal cyst included the cancer antigen 125–24.7 Units/milliliter and *Echinococcus granulosis* immunoglobulin G antibodies of 1.04 g/l*,* within the normal range. Magnetic resonance imaging (MRI) of the abdomen/pelvis at 16 weeks of gestation showed an imagistic aspect of cyst-adenofibroma of 7.3/9.5/8.4 cm on the right adnexa. We also noted hydronephrosis on the right side.

**Fig. 1:**
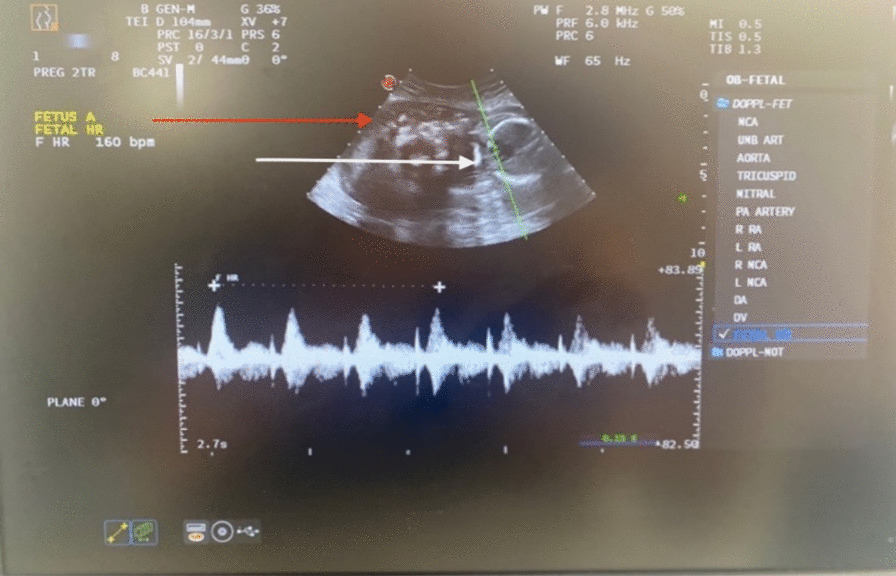
Abdominal ultrasound showed viable a fetus corresponding to 19 weeks of gestation. Presence of cardiac activity (white arrow) and multilocular cyst on the left side of the image (red arrow)

**Fig. 2:**
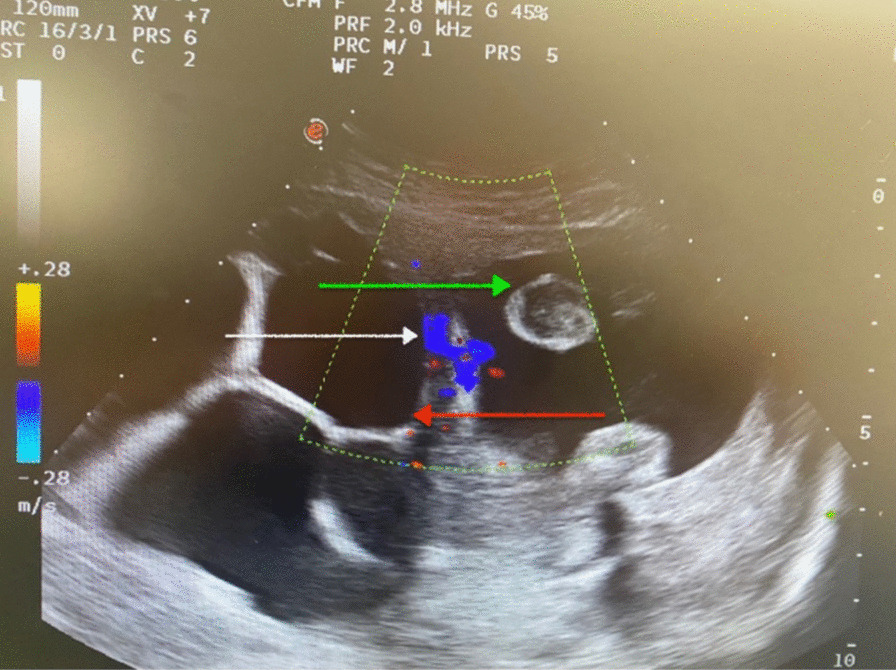
Abdominal ultrasound showed a multilocular cyst at the right side of the uterus (red arrow) with intense vascularized on Doppler ultrasound (white arrow). Thin uterine wall with fetal parts (green arrow)

At 5 weeks after the previous hospitalization, the patient returned to the hospital complaining of intense pelvic-abdominal pain and an increased temperature of 38.5 °C. The laboratory works revealed leukocytosis with 26,830 leucocytes/l, mild anemia with a hemoglobin level of 10.1 g/dl, and a thrombocytosis of 719,000 thrombocytes/l. The urine culture was again positive for *Escherichia coli*. We performed another abdominal/pelvic ultrasound, which revealed a single live fetus corresponding to 19 weeks of gestation and a multilocular cyst on the right adnexa (Figs. 1 and 2). A nephrological consult revealed a right, acute obstructive pyelonephritis and the nephrologist recommended a ureteral stenting. A ureteral stent was placed on the right ureter, which partially resolved the symptoms but increased the ureter hydronephrosis to the third degree.

**Fig. 3:**
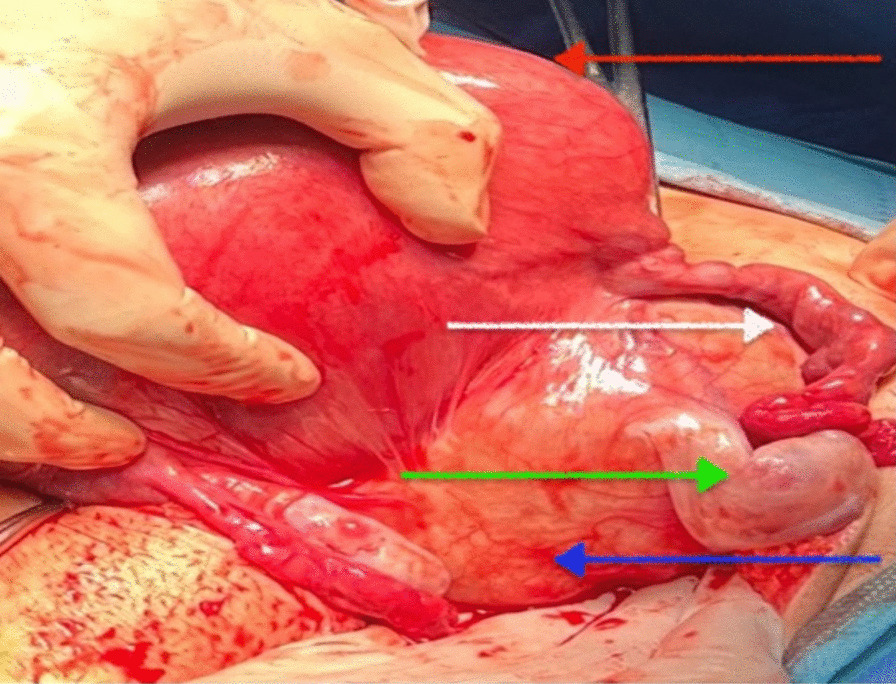
Intraoperative image of an enlarged 19-week pregnancy uterus (red arrow), a right cyst of the broad ligament (blue arrow) with normal salpinx (white arrow) and ovary (green arrow)

**Fig. 4:**
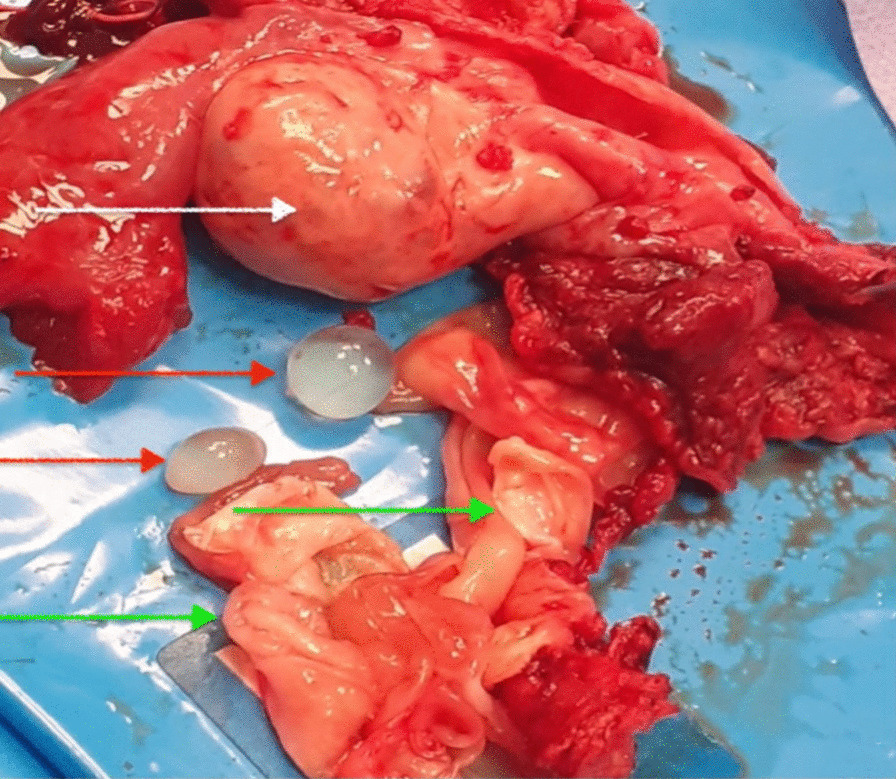
Intraoperative image of right ovary (white arrow), rupture of the cyst with multiple daughter cysts (red arrow) and proliger membrane (green arrow)

Following a collaborative meeting among the gynecologist, anesthesiologist, nephrologist, and urologist, it was determined that the cyst should be removed. The primary justification was ureter hydronephrosis and recurrent urinary tract infections caused by the cyst compression. The patient underwent an iterative median incision surgery, during which an enlarged mass of 10 × 10 × 10 cm was discovered in the broad ligament of the uterus. The mass was adhered to the posterior side of the uterus and ruptured during mobilization manoeuvres, revealing multiple daughter cysts and confirming a diagnosis of broad ligament hydatids (Figs. 3 and 4). The cyst was successfully removed, and an ultrasound examination confirmed the fetus was still alive with no signs of distress.

During the immediate postoperative period, hemoglobin was 7.2 g/dl and the patient received two units of packed red blood cells.

The patient was managed in the intermediate care unit postoperatively, and her progress was uneventful. She had severe thrombocytosis with 929,000 thrombocytes/dl and received aspirin at 100 mg/day. The infectious disease specialist introduced albendazole therapy at 400 mg/12 hours for 28 days. After 11 days of hospitalization, she was discharged with all the paraclinical investigations within normal range, including the cervical smear and the urine culture. The cutaneous scar had a slightly hypertrophic keloid aspect.

The anatomopathological examination showed a proliger membrane-hydatid cyst and acute salpingitis.

Upon her 30–31 week gestation check-up, the patient’s abdominal-pelvic exam showed normal range with typical pregnancy symptoms and no signs of cyst recurrence. Her cervix measured 2.1 cm in length, and she was prescribed dexamethasone at 6 mg/12 hours in four doses to prevent premature respiratory distress syndrome.

Then, 6 weeks later, at 37 weeks of gestation, the patient returned to the hospital owing to uterine contractions. However, her obstetrical exam showed no abnormalities, and there was no abnormal vaginal discharge or bleeding during the vaginal exam. An abdominal ultrasound revealed a single live fetus corresponding to 37 weeks of gestation, and paraclinical investigations showed a leukocytosis with 16,030 leucocytes/dl, a hemoglobin of 12.9 g/dl, and no thrombocytosis. The urine culture came back positive for *Proteus Mirabilis*, which was treated with cefuroxime 1 g/12 hours for 7 days. The patient’s ureteral stent was also removed.

**Fig. 5:**
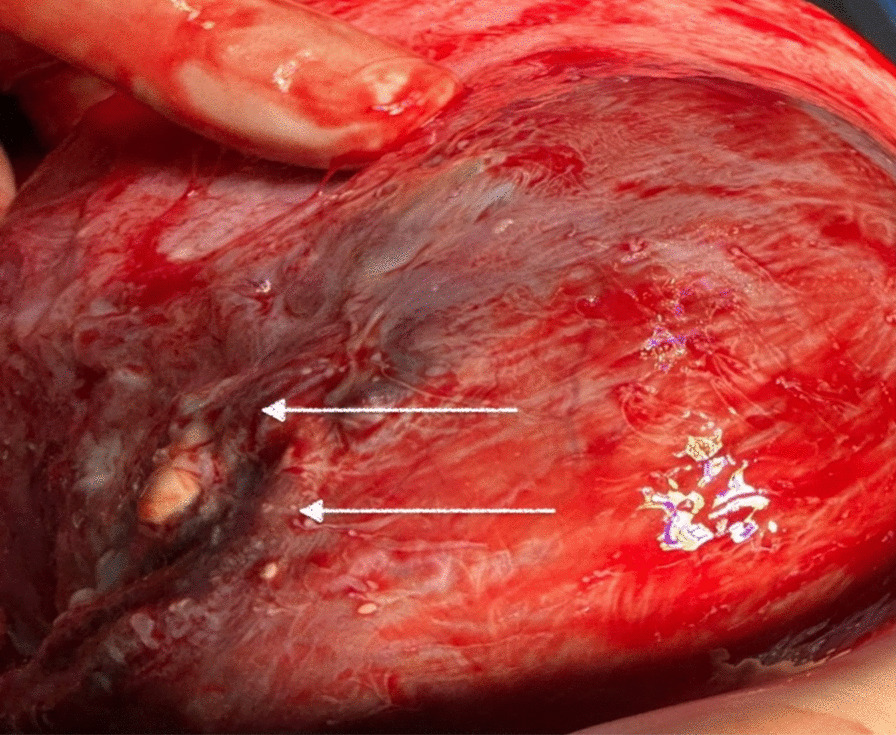
Intraoperative image of the right posterior wall of the uterus (white arrow) during the cesarean section. Adnexectomy from previous intervention with no sign of cyst recurrence

**Fig. 6:**
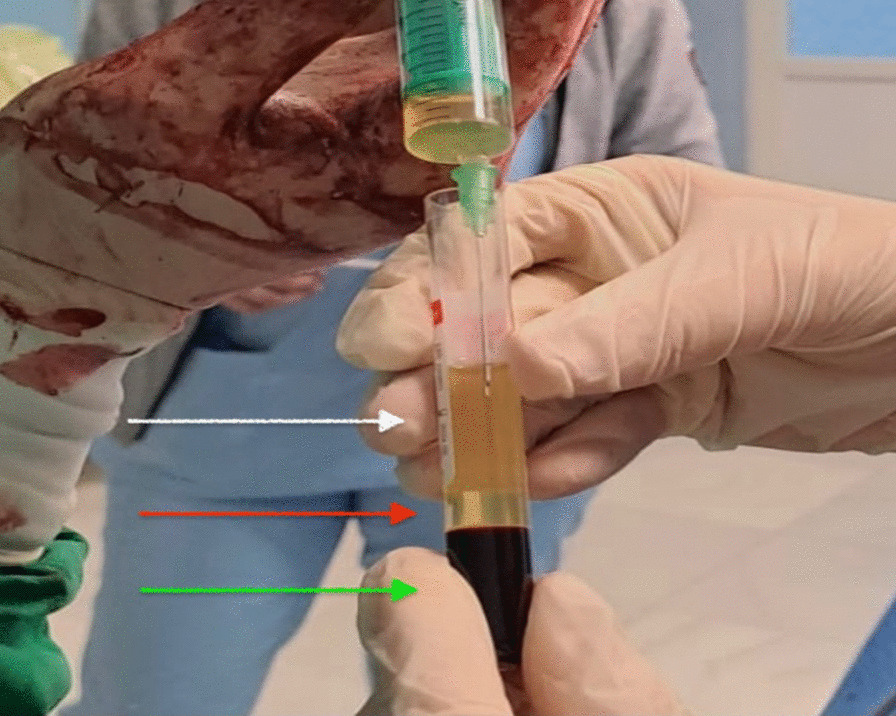
Intraoperative image of platelet-rich plasma prepared for application. There are three layers seen in the tube: the upper layer of platelet-poor plasma (white arrow), the middle layer of platelet-rich plasma (red arrow), and the bottom layer is the red blood cells (green arrow)

**Fig. 7:**
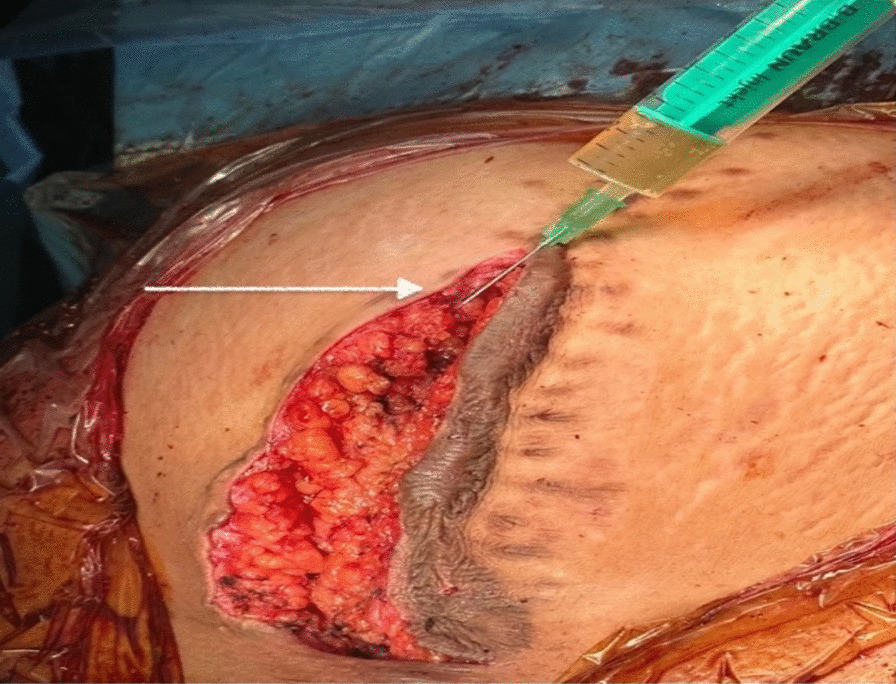
Intraoperative image of a median incision after closing the aponeurotic layer. Platelet-rich plasma is injected at the level of the previous cutaneous scar to prevent keloid scar recurrence (white arrow)

At 38 weeks of gestation, the patient underwent a lower segment cesarean section owing to fetal distress during labor. A live female baby weighing 2670 g with an APGAR Index of 9 was delivered through a midline iterative incision under spinal anesthesia. No signs of cyst recurrence were observed during the inspection of the posterior wall of the uterus and the right adnexal area (Fig. 5). Autologous platelet-rich plasma was injected in five spots, 2 ml/spot subcutaneously, and the cutaneous scar was then closed (Figs. 6 and 7). Both the patient and baby had a stable and unremarkable recovery after 10 days of hospitalization, and all paraclinical investigations, including cervical smear and urine culture, were within normal range.

**Fig. 8:**
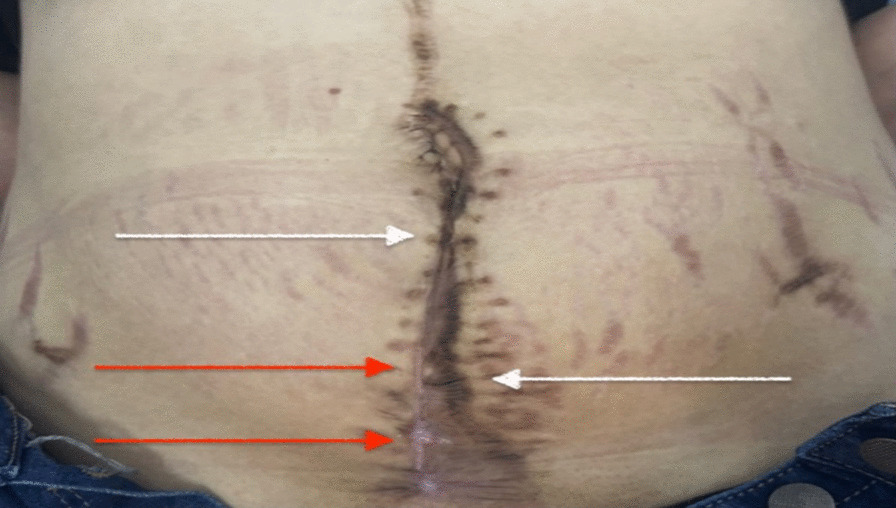
Postoperative image at 40th-day follow-up after cesarean section. The aspect was highly improved where platelet-rich plasma was applied (red arrow) in comparison with the areas where platelet-rich plasma was not applied (white arrow)

At the 40th day follow-up, transvaginal echography showed no signs of cyst recurrence, and an abdominal-renal echography showed the resolution of ureter hydronephrosis. The aspect of the cutaneous scar was highly improved where platelet-rich plasma was applied in comparison with the areas where platelet-rich plasma was not applied (Fig. 8).

## Discussion

It is extremely rare to find a hydatid cyst localized in the broad ligament of the uterus. Although hydatid disease during pregnancy is reported in about 1 in 20,000–30,000 pregnancies, localization near the genital organs is only about 0.5% of this occurrence [11, 12]. Furthermore, a study by Bickers *et al*. revealed that the incidence of hydatid cysts in the broad ligament is only 0.37% [15].

In endemic regions, risk factors for human cystic echinococcosis include free-roaming dogs, slaughtering livestock at home, or inadequately supervised slaughterhouses [16]. In this particular case, the patient was exposed to multiple dogs and sheep and could have benefited from better hygiene practices. It is likely that this cyst was a recurrence of a disseminated disease from the patient’s childhood. Unfortunately, the patient did not follow the prescribed albendazole treatment by the infectious disease doctor or undergo subsequent check-ups after the intervention.

Although imaging remains a crucial tool for diagnosing cystic echinococcosis, serological assays can also provide valuable information. However, owing to their low sensitivity (with up to 30% false negativity) and low specificity (with up to 25% false positivity), interpreting serological results can be challenging [17]. In our case, we found that the *Echinococcus granulosis* immunoglobulin G antibodies determined by enzyme-linked immunosorbent assay were within the normal range, which supported the imaging findings and led to an initial expectative management approach.

Platelet-rich plasma, which contains growth factors and platelets, has been shown to enhance wound healing and improve the quality of scar repair [18]. Studies have suggested that platelet-rich plasma can speed up healing time [19]. Furthermore, it has been found to have positive effects on wound healing and pain reduction in high-risk patients, such as those with keloid scars, who are undergoing cesarean section [20]. In our patient’s case, who had a keloid scar and underwent a cesarean section, we administered platelet-rich plasma subcutaneously during the operation to prevent the recurrence of the hypertrophic keloid aspect of the scar. At the 40th-day follow-up, we observed an improved cutaneous scar appearance compared with the previous one, thanks to the platelet-rich plasma application.

Albendazole can be used as a form of therapy for surgical and percutaneous treatment [21]. It is not recommended as the first line of treatment and is usually avoided during pregnancy, especially in the first trimester owing to the risk of teratogenicity [22]. However, inadvertent exposure to albendazole during the first trimester is unlikely to increase the risk of adverse birth outcomes [23]. We consulted with an infectious disease specialist and initiated albendazole therapy at 24 weeks of gestation, and the baby was born without any teratogenic problems.

Hydatid cysts often manifest as pelvic masses that exert pressure on nearby organs, such as the urinary bladder, rectum, uterus, ovaries, fallopian tubes, and lumbosacral nerve plexus. This can lead to clinical features, such as obstructive uropathy, which can result in renal failure or constipation [24]. Early surgical intervention for pelvic cysts that cause obstructive uropathy may prevent the progression of renal damage [25]. In our case, the enlarged pelvic mass exacerbated the renal consequences and enlarged uterus. However, after the patient gave birth, the ureter hydronephrosis was resolved, and the uterus returned to its original position as a pelvic organ.

## Conclusion

Hydatid disease represents a major health issue that requires a multidisciplinary approach and a correct fast diagnosis, especially when we talk about obstetrical patients. This medical case report provides a detailed description of a pelvic hydatid cyst that was discovered during pregnancy. What makes this case particularly noteworthy is the cyst’s unusual location—it was found at the level of the right broad ligament of the uterus that led to an even greater difficulty of care. Despite the complexity of the situation, the patient was able to receive effective treatment and the cyst was successfully managed with great outcomes for both the patient and the newborn. We hope that this report serves as a valuable example of how medical professionals can navigate challenging cases and provide optimal care for their patients.

## Data Availability

The data that support the findings of this study are available on request from the corresponding author, DB

## References

[1] Kohansal MH, Nourian A, Bafandeh S (2015). Human cystic echinococcosis in Zanjan area, northwest Iran: a retrospective hospital based survey between 2007 and 2013. Iran J Public Health.

[2] Moro P, Schantz PM (2009). Echinococcosis: a review. Int J Infect Dis.

[3] World Health Organization. Echinococcosis. https://www.who.int/news-room/fact-sheets/detail/echinococcosis. Accessed 31 Jan 2023.

[4] Centers for Disease Control and Prevention. Parasites- Echinococcosis. https://www.cdc.gov/parasites/echinococcosis/biology.html. Accessed 16 July 2023.

[5] German I, Airinei R (1994). Boala hidatică, hidatidoza, echinococoza sau chistul hidatic (Vol. Două boli parazitare grave: trichineloza și chistul hidatic).

[6] Miran MB, Kasuku AA, Swai ES (2017). Prevalence of echinococcosis and Taenia hydatigena cysticercosis in slaughtered small ruminants at the livestock-wildlife interface areas of Ngorongoro, Tanzania. Vet World.

[7] Eckert J, Deplazes P (2004). Biological, epidemiological, and clinical aspects of echinococcosis, a zoonosis of increasing concern. Clin Microbiol Rev.

[8] Larrieu EJ, Frider B (2001). Human cystic echinococcosis: contributions to the natural history of the disease. Ann Trop Med Parasitol.

[9] Ghosh JK, Goyal SK, Behera MK, Dixit VK, Jain AK (2014). Hydatid cyst of liver presented as obstructive jaundice in pregnancy; managed by PAIR. J Clin Exp Hepatol.

[10] Thompson A, Chiodini P, Stewart F (2012). Hydatid liver cyst in pregnancy: a case report. Arch Dis Child Fetal Neonatal Ed.

[11] Thakare PY (2014). Hydatid cysts in a pregnant uterus. J Obstet Gynaecol India.

[12] Başgül A, Kavak ZN, Gökaslan H, Küllü S (2002). Hydatid cyst of the uterus. Infect Dis Obstet Gynecol.

[13] Lee KW, Devaraj NK, Ching SM, Veettil SK, Hoo FK, Deuraseh I, Soo MJ (2021). Effect of SGLT-2 inhibitors on non-alcoholic fatty liver disease among patients with type 2 diabetes mellitus: systematic review with meta-analysis and trial sequential analysis of randomized clinical trials. Oman Med J.

[14] Devaraj NK (2017). Antibiotic resistance: a real menace. Oman Med J.

[15] Bickers WM (1970). Hydatid disease of the female pelvis. Am J Obstet Gynecol.

[16] Ghatee MA, Nikaein K, Taylor WR, Karamian M, Alidadi H, Kanannejad Z, Sehatpour F, Zarei F, Pouladfar G (2020). Environmental, climatic and host population risk factors of human cystic echinococcosis in southwest of Iran. BMC Public Health.

[17] Sarkari B, Rezaei Z (2015). Immunodiagnosis of human hydatid disease: where do we stand?. World J Methodol.

[18] Chaichian S, Mirgaloybayat S, Tahermanesh K, Mohammadi MH, Saadat Mostafavi R (2022). Effect of autologous platelet-rich plasma on Cesarean section scar; a randomized, double-blinded pilot study. Shiraz E-Med J.

[19] Tehranian A, Esfehani-Mehr B, Pirjani R, Rezaei N, Sadat Heidary S, Sepidarkish M (2016). Application of autologous platelet-rich plasma (PRP) on wound healing after caesarean section in high-risk patients. Iran Red Crescent Med J.

[20] Elkhouly NI, Elkilani OA, Kolaib HA, Elkhouly RM, Morsi DI (2021). Does autologous platelet-rich plasma improve wound healing and pain perception after cesarean section in high-risk patients?. Gynecol Obstet Invest.

[21] Peyvandi S, Zamaniyan M, Rahmani Z, Hoseini S (2017). Conservative treatment of hydatid cyst in pregnancy: case report. J Genit Syst Disord.

[22] Gyorkos TW, St-Denis K (2019). Systematic review of exposure to albendazole or mebendazole during pregnancy and effects on maternal and child outcomes, with particular reference to exposure in the first trimester. Int J Parasitol.

[23] Llanos O, Lee S, Salinas JL, Sanchez R (2020). A 28-year-old pregnant woman with a lung abscess and complicated pleural effusion. Chest.

[24] Kumar N, Garg R, Namdeo R (2018). Primary pelvic hydatid cyst: a rare case presenting with obstructive uropathy. Int J Surg Case Rep.

[25] Yağmur I, Kocaman OH, Dere O, Demir M, Katı B, Boleken ME (2022). Multiorgan echinococcosis with uterine involvement causing bilateral hydronephrosis in a child: case report. Iran J Parasitol.

